# The Double-Edged Sword Role of Viruses in Gastric Cancer

**DOI:** 10.3390/cancers12061680

**Published:** 2020-06-24

**Authors:** Paulina Niedźwiedzka-Rystwej, Ewelina Grywalska, Rafał Hrynkiewicz, Mikołaj Wołącewicz, Rafał Becht, Jacek Roliński

**Affiliations:** 1 Institute of Biology, University of Szczecin, Felczaka 3c, 71-412 Szczecin, Poland; rafal.hrynkiewicz@gmail.com (R.H.); mikolaj.wolacewicz@gmail.com (M.W.); 2 Department of Clinical Immunology and Immunotherapy, Medical University of Lublin, 20-093 Lublin, Poland; ewelina.grywalska@gmail.com (E.G.); jacek_rolinski@wp.pl (J.R.); 3 Clinical Department of Oncology, Chemotherapy and Cancer Immunotherapy, Pomeranian Medical University of Szczecin, 70-204 Szczecin, Poland; rbecht@pum.edu.pl

**Keywords:** gastric cancer, oncogenic viruses, oncolytic viruses, immunology, cancer treatment

## Abstract

Due to its high morbidity and mortality, gastric cancer is a topic of a great concern throughout the world. Major ways of treatment are gastrectomy and chemotherapy, unfortunately they are not always successful. In a search for more efficient therapy strategies, viruses and their potential seem to be an important issue. On one hand, several oncogenic viruses have been noticed in the case of gastric cancer, making the positive treatment even more advantageous, but on the other, viruses exist with a potential therapeutic role in this malignancy.

## 1. Introduction

Gastric cancer is, according to different sources, the 5th most common cancer in the world [1]. According to the GLOBOCAN 2018 database, over 1 million cases were reported in 2018, including 781,631 deaths [1]. However, using this database, one can notice a downward trend in the incidence of this particular type of cancer [2]. Tumors are caused not only by accidental errors in the process of DNA replication and repair, but also by the body’s exposure to harmful physical and chemical factors. Diet and activity have a significant impact on morbidity. All these factors affect a number of cytogenetic changes leading to uncontrolled cell proliferation and, as a result, the formation of tumors [1]. Viruses are known to possess oncogenic function, meaning that they are suspected of causing cancer in about every 10th case [3]. The most prominent and frequent pathogens related to cancers are human papillomavirus (HPV; associated with 640,000 cases), hepatitis B virus (HBV; 420,000 cases), hepatitis C virus (HCV; 170,000 cases) and Epstein–Barr virus (EBV; 120,000 cases) [3], but the oncogenic role of several others have also been confirmed in different types of cancer (Table 1).

Nevertheless, viruses have two faces—apart from being a cancer factor, viruses can also kill malignant cells, simultaneously sparring the healthy ones [4]. This oncolytic feature is interesting, as it potentially may be translated into clinical/therapeutic advantage, showing, that viruses have a double-sword role in gastric cancer.

## 2. Oncogenic Viruses

Several viruses are known to have a confirmed oncogenic role in different types of malignancies (Table 1). Nevertheless, there are also viruses, with only a potential oncogenic role, where studies are limited or ambiguous (Table 2).

### Oncogenic Viruses and the Immune System

The invasion of virus into the host caused a cascade of immune system actions [18]. It is worth remembering, that oncogenic viruses generally maintain chronic infections, not acute states, while the first resembling the state of carcinogenesis [19]. There are two ways of targeting the host cell ensuring cellular replication—virus may be either maintained as genetic element and viral genomes form episomes or it can integrate into the host genomic DNA [19]. In both mechanisms, a specific interaction is seen between the virus and the host cell, while oncogenic virus nurture infection of a controlled number of cells [19]. If the cancer cell dies, it will be also the end of the virus, so in a way the replication of the virus keeps both sides of the contract running. Over all, carcinogenesis is increasing, when antiviral immune responses are impaired [20]. Oncogenic viruses are also manipulating several signaling pathways, what severely interferes the actions. Main pathways are Pi-3K-AKT-mTor, MAPK, Notch, WNT-*β*-catenin and NK-*κB* [21].

Direct tumorigenesis is mediated by carcinogenic agents helping to keep the tumor phenotype and help the virus maintain as a genetic element (commonly retroviruses), while indirect transformation is conditions by two mechanisms—one is triggering chronic infection, and the second is immunosuppression (mostly presented by HBV, HVC and HIV). It is worth mentioning, that EBV, but under the same conditions also HBV and HCV, are viruses using both direct and indirect mechanism of carcinogenesis [19].

**Figure 1:**
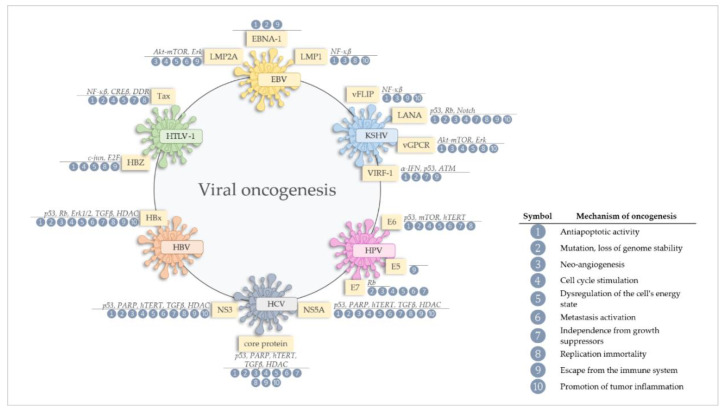
Mechanisms of oncogenesis and the involvement of viral oncoproteins in gastric cancer. All virus-associated tumors result from the cooperation of many oncogenic mechanisms. In gastric cancer, viral oncoproteins are triggering all (1–10) of the described oncogenic scenarios.

Summing up, several mechanisms are enumerated as viral oncogenic mechanisms (Figure 1), and all those mechanisms are directly or indirectly connected to different stages of the viral life cycle [19], like genomic instability, the cell proliferation, resistance to apoptosis, alterations in DNA repair mechanisms and cell polarity changes [10,19]. Viral agents also indirectly contribute to the development of cancer mainly through immunosuppression or chronic inflammation, but also through chronic antigenic stimulation. There is also evidence that viruses can modulate the malignant properties of an established tumor [19]. Moreover, one of the strategies to avoid antiviral immunity by oncogenic viruses (DNA and RNA) is the ability to regulate host DNA methylation [20]. Inducing hypermethylation of immune genes is leading to viral replication and persistence and is a common mechanism to potentiate virus-induced cancer progression [20]. An important factor impacting oncogenesis may also by miRNA, participating in cell transformation, by inhibiting mRNA translation [19,22]. 

## 3. Oncogenic Viruses in Gastric Cancer

### 3.1. Epstein–Barr Virus

The Epstein–Barr virus is one of the human herpesviruses with a proved oncogenic potential [1]. It belongs to the Herpesviridae family in the Herpesvirales order [23]. It has linear double-stranded DNA 168–184 kbp long, which consists of 85 genes [24]. Due to the difference in the EBNA gene, 2 subtypes of EBV 1 and 2 were distinguished [1,24,25].

EBV, like all herpesviruses, has a latent and lytic phase [26]. The infection of B lymphocytes with EBV in cell culture results in the establishment of an immortalized B cell line [27]. There are several proteins encoded in the EBV genome that have transformational potential. One of them is LMP1 (latent membrane protein), which has the ability to transform equal types of cells, including fibroblasts in rodents [28]. In addition, the LMP1 gene is necessary for the virus to kill B lymphocytes, since its removal causes a lack of transformation [28].

LMP1 has many transmembrane spanning domains and its carboxyl terminus may interact with several tumor necrosis factor receptor associated factors (TRAF) [26,29]. The interaction between LMP1 and TRAF results in high expression of the nuclear factor kB (NF-kB) in LMP1-expressing epithelial and B cells [26]. LMP1 also upregulates the expression of some genes responsible for apoptosis and adhesion, including A20, bcl2 and ICAM-1 [26]. In addition, it activates the expression of interferon regulatory factor 7 (IRF-7) [30], matrix metalloproteinase 9 (MMP-9) and fibroblast growth factor-2 (FGF-2) [31].

Another viral gene, LMP2, has been shown to inhibit B-cell receptor (BCR) signaling [32]. It works by sequestering the Src family members Fyn and Lyn, preventing their translocation into lipid rafts with BCR, thereby inhibiting BCR activity [33].

Other viral genes that encode transforming potential include EBV nuclear antigen 2 and 3 (EBNA2 and EBNA3). EBNA2, like LMP1, is necessary for the transformation of B cells, because the removal of this gene from the wild type EBV makes the virus unable to kill B cells [26,34]. Among the genes encoding EBNA3, EBNA3A and EBNA3C, they are necessary for the transformation of B cells, while EBNA3B is unnecessary [35]. All three EBNA3 proteins can interfere with EBNA2 activation, interfering with its intercalation with RBP-Jk DNA-binding protein, thereby suppressing its EBNA2-mediated transactivation [26]. EBNA3C may therefore promote cell proliferation and cross the G1-S phase checkpoint and may also work with EBNA2 and EBNA3A to modulate cell gene expression in EBV infected lymphocytes.

In general, the oncogenic mechanism of EBV relies on coding LMP1 and LMP2, EBNA1-3, leader protein (LP), BamHI A reading frame 1 (BARF1) and BamHI A rightward transcript miRNAs, which their role is to promote transformation of B cells and epithelial cells and block pro-apoptotic proteins in host cells [20]. Lately it was also confirmed [20] that stimulation of DNA hypermethylation of host genes might contribute to carcinogenesis.

It is widely believed that EBV contributes to the development of many diseases, including Burkitt’s lymphoma, Hodgkin’s lymphoma, diffuse large B-cell lymphoma, lymphoproliferative disorder in people with immunodeficiency [24,36,37], post-transplant lymphoproliferative disease, central nervous system lymphoma, non-Hodgkin lymphoma, oral hairy leukoplakia [38] and gastric cancer [24,38,39,40,41,42,43,44].

Many different independent studies confirm the presence of EBV virus in cancer cells, among others, in lymphoepigastric adenocarcinomas [40], lymphoepithelioma-like gastric carcinoma with marked lymphocytic stroma [41]. In general, EBV is detected in approximately 10% of gastric cancer cases [42,45]. Its existence in gastric cancer was first discovered in 1990, by the means of a polymerase chain reaction (PCR) [46]. The EBV-encoded small RNA 1 (EBER1) gene is used to confirm the presence of the virus in cancer cells. It is a viral protein attributed to the function of combining viral DNA with host chromosomes, which enables its replication by host DNA polymerase [24]. The presence of this gene can be confirmed by carrying out both the polymerase reaction and in situ hybridization (ISH) [44]. When the ISH method is used, EBER1 signals are detected in the nuclei of gastric cancer cells [41,42,47]. In EBV positive stomach cancer (EBVaGC), all cancer cells carry the EBER1 gene [43].

Published in 2014, the Cancer Genome Atlas (TCAG) study, presented the gastric adenocarcinomas division into four groups: 1. EBVaGC; 2. microsatellite instability (MSI); 3. chromosomal instability (CIN) and 4. genomically stable (GS) tumors [48]. It has been reported that EBV 16 positive tumors are characterized by the transmission of recurrent mutations in the PIK3CA gene, DNA hypermethylation and overexpression of the JAK2, PD-L1 and PD-L2 genes [38,43,48,49].

In 2015, Chen et al. [44] published a systematic review in which they focused not only on studies showing the presence of the EBER1 gene in cancer cells and also in non-tumor tissues adjacent to the stomach in cancer patients, in the non-tumor mucosa of healthy patients, patients with mild stomach diseases and in the deceased individuals and studies comparing anti-EBV antibodies in the serum of healthy and sick patients. They analyzed 47 studies; in total 9909 patients were examined, including 8069 patients and 1840 healthy people. The EBER1 positivity tested by the ISH method was significantly higher and ranged from 5% to 17.9% in the tumor tissue than in the adjacent mucosa in the same patients or biopsies from all control groups—almost 0%. They also noted that some cases of confirmation of the presence of EBER1 by PCR were not confirmed by ISH. They concluded that the ISH method makes it possible to effectively determine the relationship between gastric cancer and EBV infection, and the PCR method is not efficient enough.

### 3.2. HHV-8

Human herpesvirus 8, like EBV, belongs to the Herpesvirales family, to the subfamily Gammaherpesvirinae [23]. It was first discovered in AIDS-related Kaposi’s sarcoma in 1994, which owes its second name: Kaposi sarcoma herpes virus (KSHV) [50]. KSHV is also involved in the development of primary effusion lymphoma, multicentric Castleman’s disease (MCD) [24,51,52] and B-cell lymphoproliferative disorders that can be converted to KSHV-associated non-Hodgkin’s lymphoma and also primary effusion lymphoma (PEL) [39,53].

The KSHV genome contains a variety of genes responsible for transformation, signaling, prevention of apoptosis and avoidance of immunity. Researchers believe that HHV-8 transforms cells through a paracrine mechanism because several studies have shown high levels of cytokines and growth factors in KS and MCD changes [26].

KSHV can immortalize primary bone marrow endothelial cells and induce cell proliferation, anchoring independence and survival of these cells. Researchers also found that only a subset of transformed endothelial cells contained viral DNA, which firmly said that adjacent uninfected cells survived due to a mechanism involving cytokines secreted by infected cells [54]. On this basis, it has been suggested that transformation of KSHV is dependent on paracrine factors [26].

The KSHV K1 genes and viral G-protein-coupled receptors (vGPCR) have oncogenic potential. The K1 protein is able to transform rodent fibroblasts in vitro, and when injected into nude mice, these cells induce numerous and widespread tumors. In addition, K1 has the ability to functionally replace the saimiri transforming protein (STP) of herpesvirus saimiri (HVS) in vitro and in vivo to induce lymphoma in marmoset monkeys [26]. Transgenic animals expressing K1 develop sarcomas and lymphomas [55]. In addition, K1 can induce B cell signaling and proliferation through an immunoreceptor tyrosine-based activation motif (ITAM) and blocking Fas-induced apoptosis of these cells [56,57]. In addition, Wang et al. found that K1 can activate the NF-kB and PI3K paths. In the endothelial cells, researchers showed that K1 upregulates the expression and secretion of vascular endothelial growth factor (VEGF) and MMP-9 [58,59].

Similarly to the K1 protein, the KSHV vGPCR protein works, which has the ability to transform NIH 3T3 cells in vitro. vGPCR can also activate phospholipase C (PLC) and PI3K pathways [60]. This protein also immortalizes primary endothelial cells and transgenic mice expressing vGPCR develop angioproliferative changes similar to Kapossi sarcoma-like lesions [61]. In addition, expression of vGPCR in various cell types leads to upregulation of many cytokines and paracrine factors. Thus, this specific viral protein may be involved in the development of KSHV-related cancer by inducing and supporting cell proliferation.

In addition to the two proteins mentioned above, the KSHV genome also encodes: interferon 1 regulatory factor (vIRF-1) and the Kaposin/K12 gene. Both of these proteins have in vitro transformation potential [26]. In addition, researchers have shown that LANA (latency-associated nuclear antigen) immortalizes endothelial cells and induces B cell and lymphoma hyperplasia in mice [62,63].

Despite learning about many models of KSHV transformation and oncogenesis, the origin of KS-related tumor cells remains controversial [64]. In order to understand the exact mechanism of KSHV oncogenesis, further research is needed, both in human and animal models, because more transformation pathways than presented may exist.

KSHV cannot transform any cells in culture and does not sustain its own persistence without EBV co-infection [39,65]. In the case of the PEL, the researchers found that KSHV/EBV co-infection occurred in most of the cases [66]. The role of EBV in this disease is not fully understood. It is believed that in this very case EBNA1 gene expression increases KSHV virus load and an increase in the extent of LANA [66]. It is possible that the function of the EBV in KSHV/EBV co-infection in other cases is also to enhance the virulence and the KSHV genome expression in the host cells.

This virus has a long dsDNA chain (over 140 kbp) [24]. Unlike EBV, KSHV does not connect to chromosomes, but connects to genomic DNA indirectly, due to the LANA1 protein with histones H2A and H2B [67].

### 3.3. Human Papillomavirus

Human papillomavirus (HPV) belongs to the Papillomaviridae family [23]. Among the many distinguished types of HPV, type 16, 18, 33, 45, 52 and 58 are associated with various types of cancer, including cervical, anogenital, penile and nasopharyngeal cancers [24,68,69,70]. Research on the potential role and development of HPV-16 and HPV-18 viruses in cervical cancer was initiated by zur Hausen et al. in the 1970s [71,72,73], for which he was awarded the Nobel Prize in Medicine and Physiology in 2008 [74]. This shows how important it is to study the role of oncoviruses, not only HPV, in order to fully understand their mechanisms of action, develop methods for their detection and discover effective treatment methods.

The HPV genome is built of 7–8 kbp circular double-stranded DNA [75]. After many studies, it has finally been determined that the most common route of transmission of this virus is the sexual route [76]. However, HPV is a very stable virus and can survive on surfaces for up to several days. The virus is also resistant to some disinfectants [76,77,78]. For this reason, the virus can also be transmitted through by non-sexual means: either by way of mother to child, fomites, self-inoculation or nosocomial infection [76]. It is very possible that all the HPV transmission routes have not yet been discovered. For this purpose, long-term prospective studies should be undertaken. Although the sexual route is the most common way it is very necessary to spread among the public about alternate modes of transmission [79].

Primary HPV infection occurs in basal epithelial stem cells [26]. Then the virus traverses upwards and replicates in finally differentiated keratinocytes, and is shed from the stratum corneum [26,80]. The HPV genome lacks an enzyme necessary for replication—DNA polymerase—and therefore the replication of the viral genome depends on the stimulation of cellular DNA synthesis in infected cells [26].

In the vast majority of cervical cancers, HPV integrates with the host genome, resulting in loss of expression of the E2 viral gene, which is the transcriptional repressor of the E6 and E7 genes. As a result, there is an increased expression of oncoproteins encoded by these two genes [80]. E6 and E7 proteins from high-risk virus strains have strong transformational abilities. It has been shown [26,81,82] that these proteins immortalize cells in vitro and induce skin tumors in transgenic animals.

HPV viral oncoproteins attack tumor suppressors. The overall result is cell cycle and cell growth dysregulation and the inhibition of the apoptosis. E6 binds the p53 transcription factor and induces its degradation [83]. E6 binds to ubiquitin ligase forming the E6-AP complex, binding p53 and causing ubiquitination and proteosomal destruction of proteins [84]. In addition, E6 may induce telomerase activity and lead to cell immortalization [85].

E7 binds members of the retinoblastoma (Rb) family [86]. This protein hinders Rb function and allows cells to enter the S phase of the cell cycle. E7 binds to the hypophosphorylated form of Rb and prevents its binding to E2F transcription factor. Free E2F transcription factors promote the expression of genes required for cell DNA synthesis, thereby pushing the cell into the cell cycle [26,87]. In addition, E7 stimulates cyclin-A and cyclin-E dependent kinase activity and deactivates p21/WAF1 and p27/KIP1 kinase inhibitors. E7 may also be the cause of the synthesis of abnormal centrioles and aneuploidy at an early stage of the oncogenic process [26].

Treatment of the effects of his infection is possible due to the discovery of HPV vaccines. Available vaccines protect against two, four or nine types of HPV, but each of them is directed at least to HPV-16 and HPV-18—the types of virus whose infection causes the greatest risk of developing cervical cancer [88].

In 2018, de Souza et al. conducted studies aimed at demonstrating correlations in co-infection with HPV, EBV and *Helicobacter (H.) pylori* in gastric cancer [13]. Three hundred and two samples were tested, most of which (55%) were classified as an enteric subtype. All three pathogens were found in the samples tested, including *H. pylori* in 87%, EBV in 20% and HPV in 3%. Interestingly, among HPV-positive samples, researchers found only viruses of Types 16 and 18. Based on the research, they concluded that human papillomavirus is not involved in the development of gastric cancer [13].

No other studies were found that clearly and undeniable confirm the correlation between HPV infection and gastric cancer.

### 3.4. Hepatitis B Virus

The hepatitis B virus (HBV) is a human, partially double-stranded DNA virus with a diameter of 42–47 nm and a genome of about 3.2 kbp [19,89]. It belongs to the family of Hepadnaviridae of the genus *Orthohepadnavirus* [23]. It is replicated in hepatocytes via indirect RNA using viral reverse transcriptase [90]. The virus has a natural tropism to the liver and in most cases, the infection leads to liver damage, with the consequence that hepatocellular carcinoma develops [91,92].

The HBV genome has a small HBx region that plays an important role in oncogenesis [93]. The HBx is a relatively small 17kDa polypeptide [94,95]. The HBx activates many different promoter elements. It is responsible for activating transcription of viral and cellular genes. It changes signal transduction, disrupting the signaling cascade above the transcription complex. These signaling cascades trigger the activation of many factors such as AP-1, NF-kB, SP1 and Oct-1 [96]. The HBx protein stimulates entry into the cell cycle by activating selected cyclins and cyclin-dependent kinase pathways, as well as pathways such as Wnt, ras, PI3K, JAK/STAT, NF-kB and Hedgehog, which promote survival and growth [97,98]. Nuclear HBx affects transcription regulation by activating CREB, ATF-2, ATF-3, NFAT, C/EBPβ and SMAD4 complexes and facilitates the introduction of epigenetic changes that affect the expression of the host cell gene [99,100]. Changes in HBx-mediated miRNA levels both modulate the expression of oncogene and the suppressor gene [101]. Hepatitis B virus avoids both growth suppression and immune destruction by blocking the process of apoptosis. Internal apoptosis is caused by the occurrence of oxidative stress caused by the virus itself, while external apoptosis is activated through the immune system [98]. The HBx blocks the activation of the key mediator of congenital antiviral signaling, which is the MAVS (mitochondrial antiviral signaling protein), while the prevention of external apoptosis is caused by TNFα, TGFβ and Fas by blocking caspases 8 and 3 and activating NF-kB, the latter being responsible for liver protection [98]. The HBx protein replaces the negative regulation of TGFβ growth and converts it into a tumor promoter [100]. Mitochondrial-associated HBx causes an increase in reactive oxygen species (ROS). High levels of ROS, combined with the progression of the cell cycle, increase the risk of occurrence and spread of mutations many times over [100]. The HBx promotes cell division by directly interacting with the p53 protein by suppressing binding and transcriptional down regulation, in addition it promotes Rb inactivation and down regulates some cdk inhibitors [96,98]. HBx also inhibits DNA 1 binding protein (DDB1) damage during repair of nucleotide excision, and also promotes the appearance of multinucleated cells, chromosome rearrangement and micronucleus formation [100]. In cell culture experiments, indeed, HBx expression significantly inhibited the ability of cells to repair damaged DNA [102]. The HBx can also cause increased angiogenesis and metastasis, through the transcription factor HIF1α, which activates Ang-2 (angiopoietin-2) and VEGF (vascular endothelial growth factor) [100]. The HBx protein influences the development of all key features of cancer and does not have to interact with other viral oncogenes [96].

Chronic HBV infection is associated with EHC (extra-hepatic cancers) such as pancreatic cancer, non-Hodgkin’s lymphoma and gastric cancer. Hepatitis B virus, through the bloodstream, can lead to infection of tissues of organs other than the liver. HBV antigens outside the liver are also often detected in the stomach, gastrointestinal tract, pancreas and kidney [92,103,104]. It is possible that HBV can replicate in extra-hepatic tissues and plays an oncogenic role [92]. Over the past several years, there have been many independent studies showing the relationship between HBV surface antigen (HBsAg) and gastric cancer [91,92,103,104,105,106,107].

Chen et al. [106] in 2004 noticed that very often a co-infection of the hepatitis B virus and *Helicobacter (H.) pylori* was observed in patients. The study involved 72 patients of Jiangsu Province in China, including 28 patients with diagnosed chronic hepatitis B and 44 patients with advanced hepatic cirrhosis caused by hepatitis B who were the study group. Thirty patients with gastritis but no liver disease was included in the control group [106]. There was no significant difference between the cirrhosis group and the group with chronic hepatitis. It was observed that HBV antigen expression in the gastric mucosa with positive *H. pylori* infection was 69.8% and with negative 73.7% (*p* > 0.005) [106]. It has been concluded that HBsAg and HBcAg overexpression coexist with *H. pylori* antigen expression in the gastric mucosa of persons with *H. pylori* infection, thus early treatment of *H. pylori* infection may be beneficial for the prognosis of patients with chronic liver disease [106]. In 2011, research was carried out in China in which ten commonly occurring extrahepatic tumors were assessed. The tests evaluated the presence of HBsAg in cancerous tissues. Approximately 14% of patients with confirmed gastric cancer received a positive result for the presence of the hepatitis B surface antigen (HBsAg) [92,107]. In contrast, in the Republic of Korea, the presence of HBsAg was confirmed in 3.4% of women and 4.7% of men with stomach cancer [92,103].

Ghasemi et al. [104] in 2012 presented their research, in which they examined the effect of HBV on gastric cancer in the population of Northern Iran. Researchers collected 100 biopsy blocks with paraffin fixed in formalin and gastric cancer was confirmed in all trials [104]. In the study group, 69% of patients with gastric cancer were middle-aged men and 31% were women. The authors did not show the presence of the HBV genome in gastric cancer in their studies, which indicates that HBV is not correlated with the development of gastric cancer in the inhabitants of Northern Iran [104].

The first studies that showed the actual relationship between hepatitis B virus infection and gastric cancer appeared in 2015. Wei et al. [105] conducted a retrospective follow-up study with 580 cases and 580 controls that matched each other by age, gender and year of diagnosis. The relationship between gastric cancer and HBV infection was investigated using one- and multi-dimensional unconditional logistic regression analysis. The results obtained show that the HBsAg antigen is positively associated with gastric cancer (AOR (95% CI): 1.49 (1.06–2.10)) [105]. However, the relationship remained significant in patients with no family history of cancer (AOR (95% CI): (1.06–2.11)). In the group with negative HBsAg, which are anti-HBc positive/anti-HBs negative, which probably suggested latent HBV infection, also shows some association with gastric cancer. Besides, some synergistic effects have been demonstrated between HBV infection and blood group A in gastric cancer. Studies directly show that HBV infection is positively associated with the development of gastric cancer, especially in a group of patients who have no confirmed family history of gastric cancer. Wei et al. stated that further prospective studies are needed to finally confirm the association of HBV with gastric cancer [105].

In 2019, the latest research appeared that aimed to confirm the relationship between HBV infection and gastric cancer [92]. The correlation between gastric pathology and hepatitis B virus infection in patients with positive or negative *H. pylori* infection was evaluated. The study involved 728 patients who underwent endoscopy in 2017–2018. Histopathological analysis of tissues was performed on samples taken from the stomach [92]. The presence of HBsAg in the serum of the examined patients was confirmed by the immunoenzymatic method (ELISA). The relationship between gastric cancer and HBV infection was examined using logistic regression analysis. From the results obtained, it appears that among 728 patients, HBsAg infection was detected in 83 (11.4%), while *H. pylori* infection was confirmed in 408 (56%) patients. Co-infection with *H. pylori*/HBV was confirmed in 69 (9.5%) patients [92]. *Helicobacter pylori* infection was significantly more frequently detected in patients with positive HBsAg than negative (*p* = 0.029) [92]. Not a single patient co-infected with *H. pylori*/HBV had normal stomach tissue. There was a significant histopathological difference in gastric tissue between patients with HBsAg positive and no *H. pylori* infection (*p* < 0.0001). The hepatitis B surface antigen (HBsAg) was associated with histopathological changes in stomach tissue (OR = 21.56, 95%CI = 7.070 − 65.741, *p* < 0.001) and may be a potential risk factor for gastritis (OR = 12.457, 95% CI = 3.007-51.614, *p* = 0.001) [92]. The effect of HBsAg infection on the development of stomach cancer was not confirmed (OR = 2.127, 95%CI = 0.242–18.704, *p* = 0.496). Baghbanian et al. [92] concluded that HBV infection alone may be associated with some precancerous lesions, but is not correlated with gastric cancer. In contrast, the hepatitis B virus, in the case of people with *Helicobacter pylori* infection, can significantly affect the severity of precancerous conditions or the development of gastric cancer [92].

### 3.5. Hepatitis C Virus

Hepatitis C virus (HCV) is a human, single-stranded, linear RNA virus with positive (+) ssRNA polarity and a length of about 9.6 kbp [108,109,110]. The virus belongs to the family Flaviviridae of the genus *Hepacivirus* [23]. It is estimated that around 171 million people worldwide are constantly infected with HCV, which causes a number of chronic liver diseases [110,111]. Hepatitis C virus, like hepatitis B virus, has a natural tropism to the liver and contributes to hepatocellular carcinoma (HCC) and gallbladder cancer [8,110].

In the case of HCV, both the core and the unstructured protein 5A (NS5A) and NS3 directly promote the development of hepatocellular carcinoma, by altering the expression of the host gene, and inflammation caused by the immune system indirectly affects the formation of tumors [112,113]. The HCV core and the NS3 and NS5A proteins promote the proliferation of liver cells through the β-catenin pathway. The core protein affects the expression of cyclin-dependent kinase 2 (cdk2) and cyclin E [100,114].

The HCV, as with the HBV, avoids growth suppression and immune destruction by inhibiting the apoptosis process [114]. HCV infection induces innate immunity, but viral proteins effectively block signaling that triggers IFNβ (interferon beta) as well as IFNα (interferon alpha) signaling by targeting JAK/STAT [100]. The HCV core and NS3 protein inactivate many suppressor genes [100]. The core blocks the process of apoptosis by inhibiting caspase 8 using the host’s immune system [100,112,114]. Binding of NS5A to signaling cellular molecules inhibits the immune response, suppressor genes and apoptosis [98,115]. The HCV virus up-regulates miR-181, which causes the appearance of “stemness” markers in hepatocellular carcinoma [100]. One of the features of HCV-associated cancers is replication immortality. In the case of hepatitis C virus, stable transfection of human hepatocytes with the HCV core promotes differentiation, continuous growth and increased expression of telomerase, which can largely promote immortality [100].

The HCV has the ability to trigger an angiogenesis process. This process is caused by the production of a large amount of ROS, which affect the activation of a number of HIF1α stabilizing signaling pathways [115].

Malignant strains of HCC have a predisposition to invade and metastasize. This is caused by elevated levels of the HGF receptor (hepatocyte growth factor) and c-met. These factors can lead to diffusion, angiogenesis, proliferation and increased cellular motility, eventually to invasion and metastasis [100]. The HCV core causes EMT (epithelial–mesenchymal transition) and tumor invasion. By activating JNK/pSmad3L signaling, the core protein abolishes TGFβ-dependent tumor suppression [115].

DNA repair and apoptosis are also regulated by poly (ADP-ribose) polymerase 1 (PARP-1). The NS5A protein stabilizes PARP-1 levels by blocking caspase 3-mediated cleavage. These processes may allow mutation reproduction and genetic instability in cells infected with HCV [100,115].

Patients infected with HCV have a higher risk of developing hepatocellular carcinoma compared to those who are not infected. Unlike HBV, which has the ability to integrate into the host genome, thereby causing direct carcinogenic activation, it is known that HCV is an RNA virus that has limited ability to integrate its genetic information into the host genome. Therefore, hepatitis c virus carcinogenicity is associated with indirect mechanisms [116]. In total, HCV and HBV caused 433,186 new liver cancer cases and 406,779 deaths in 2012, which is 77.61% of liver cancer cases and 76.6% of deaths [8]. There are no data to date regarding the impact of HCV on the development of gastric cancer.

### 3.6. HTLV-1

Human T-lymphocytes lymphoma virus-1 is a member of Retroviridae [23]. The association of human T-cell lymphotroptic virus 1 with cancer is controversial, and positive correlation was confirmed in case of human leukemia [117,118]. Transmission of the virus is possible through sexual intercourse, breastfeeding and contaminated blood [119]. First line of infection with the virus are DCs (dendritic cells), afterwards HTLV-1 may be transmitted to CD4^+^ T cells and, to a lesser extent, CD8^+^ lymphocytes, B cells and monocytes [119]. It is worth mentioning that viral spread of HTLV-1 is dependent on cell-to-cell contact, but also forming a viral biofilm or virological synapse [120,121]. Other possible spreading mechanisms may include nanotubes [122]. The latest data show that the promotion of cell-to-cell contact may be influenced by the formation of extracellular vesicles, to elicit adverse effects on recipient uninfected cells [123].

Oncogenic mechanisms are not evident for HTLV-1, nevertheless, a crucial role may be performed by the regulatory proteins Tax and HBZ with oncogenic properties [119,124]. Tax is a trans-acing viral protein being a major target of CTLs (cytotoxic lymphocytes), and its mechanism of transformation is related to reprogramming cell cycle and the inhibition of DNA repair [19], while HBZ is a leucine zipper factor with low immunogenicity, suppressing major HTLV-1 genes, possessing a role in cell proliferation, apoptosis, T-cell differentiation and immune escape [125]. Moreover, the differences in oncogenic mechanism may also result from the alterations of the infected cell microenvironment [123]. Additionally, similarly to other oncogenic viruses, the infection favors chronic infection, leading to immunosuppression and cancer development [124]. In blood malignancies it was confirmed, that one of the oncogenic mechanisms involving HTLV-1 infection is the dysregulation of gene expression, leading to abnormal chromatin looping, changing the position of HTLV-1 promoter-enhancer position [126].

Studies have been performed in the association of HTLV-1 with gastric cancer, leading to the conclusion, that the prevalence of HTLV-1 infection in patients with gastric cancer appears to be significantly lower than that in control patients [127,128,129]. Additionally, HTLV-1 reduces the risk of *Helicobacter pylori* infection, thus indirectly, influences the lower rate of gastric cancer, as *H. pylori* infection is known to be a frequent risk factor of this type of cancer [128].

### 3.7. Human Immunodeficiency Virus

Human immunodeficiency virus (HIV) is single-stranded RNA virus with positive (+) ssRNA polarity [130], belonging to the family Retroviridae of the genus *Lentivirus* [23]. So far, two types of HIV have been distinguished—HIV-1 and HIV-2 [130]. Both types of virus evolved from two different viruses attacking monkeys—SIV (Simian immunodeficiency virus) [130].

HIV infection leads to serious changes that disrupt the immune system of the host, making it extremely susceptible to other viral, bacterial and fungal infections. This condition was called acquired immune deficiency syndrome—AIDS [131]. Most often, HIV infection occurs through sexual contact, but also this virus can get into the bloodstream through contaminated needles or perinatally from an infected mother [131]. In most cases, untreated HIV infections lead to death [130,131]. The HIV virus, by weakening the host’s immune system, reduces the body’s ability to defend itself effectively and combat viral infections leading to the development of cancer [132]. An organism without defense becomes an excellent environment that allows the free development of other oncogenic viruses such as KSHV, EBV, HPV, HBV, HCV and HTLV-1 [133].

The HIV virus may have oncogenic potential through direct cellular mechanisms mediated primarily by the HIV Tat protein [134]. The Tat protein is an early unstructured protein, essential for viral replication [135]. Tat HIV is released from HIV-1 infected cells. The protein can bind to uninfected cells, including endothelial cells, and infiltrate through the domain of protein transduction [136]. The Tat HIV protein can affect the blocking of the tumor suppressor gene function as well as activate proto-oncogenes, inhibit apoptosis and affect cell cycle progression [134].

People infected with HIV have a higher risk of certain types of cancer than people of the same age who have not been found to be infected with HIV [137]. The HIV virus mainly contributes to the development of Kaposi’s sarcoma, aggressive B-cell non-Hodgkin’s lymphoma and cervical cancer [132]. These cancers are referred to as “acquired immunodeficiency syndrome (AIDs)-defining cancers” or “AIDS-defining malignancies”. Diagnosing each of these three cancers in people who are positive for HIV confirms the diagnosis of AIDS [132].

In addition, it has been observed that HIV infection can lead to the development of other cancers known as non-AIDS-defining cancers, such as cancers of the anus, penis, liver, oral cavity/pharynx and lung, and Hodgkin lymphoma [137,138,139,140].

To date, no confirmed involvement of HIV-1 in gastric cancer has been reported. In 2012, Perrson et al. presented the study, which showed that the risk of gastric cancer was significantly increased among patients with confirmed AIDS (SIR = 1.44; 95% CI, 1.17–1.76) [141]. For the general population, the incidence rate for stomach cancer was 5.00 per 100,000 person-years [141]. Unfortunately, in 2016, this article was withdrawn due to irregularities in statistical surveys. As a result of errors, standardized incidence ratios (SIRs) were too high. SIR corrected results are lower than the authors reported, and corrected SIR for gastric cancer is no longer significantly increased [142].

## 4. Adenovirus—Oncogenic or Oncolytic?

The role of adenoviruses in gastric cancer is mysterious. On one hand, adenoviruses (Adenoviridae) [23] are known to be oncogenic in many malignances [143,144], but the oncolytic properties of the virus also exist. In the case of gastric cancer, only oncolytic properties have been used in several studies in order to improve the possibilities of therapies. For effective oncolytic activity, adenoviruses must specifically infect and replicate within cancer cells, but unfortunately, many malignant cells do not express the CAR receptor (coxsackie and adenovirus receptor), resulting in decreased transduction of serotype 5 Ad (Ad5), which is commonly used for Ad-based vectors [145]. Therefore, efforts are made, to modify Ad5 fibers, the capsid moiety responsible for virus–cell surface receptor interaction, in order to increase their transduction to cancer cells [145]. It is also known, that adenovirus vector has a capacity to produce high titers and is genomically stable, with a low rate of DNA integration into the host’s genome [146]. On the other hand, adenovirus vectors may induce potent immunogenic toxicities, followed by the inhibition of the expression of transgene mediated by the vector itself, leading to several limitations of this kind on cancer therapy, so the good and the bad face of the virus treatment is here also as an issue [147]. In gastric cancer, adenovirus vectors have also been used [148]. There are reports about oncolytic adenoviral vectors, in which modification has been made, by replacing E1a and E1b promoters of adenovirus with human telomerase reverse transcriptase (hTERT) and hypoxia response element (HRE) promoters, leading to creation of recombinant oncolytic adenovirus KGHV [148]. The study by Wang et al. [148] shows that the infection of normal cells may be decreased by the combination of KGHV500 adenovirus, targeted at CIK (cytokine-induced killer cells), with a known anticancer potential [148]. This leads to the conclusion, that such way of delivering oncolytic viruses to tumor targets are a promising method.

Additionally, tumor-specific midkine and cyclooxygenases (Cox2M and Cox2L) promoters were tested in gastric cancer, and high activity was noted with oncolytic effect was confirmed for Cox2CR-Ad complex and fiber-modified vector Ad5/3 [149].

There is also a study showing, that adenovirus bound as a vector with Arg-Gly-Asp peptides in the fiber knob, allowed the virus to utilize integrins, which is a very promising target, while integrins are highly expressed in gastric cancer [143].

In another study, a CEA promoter was introduced into an adenovirus vector, and this method was also a successful attempt at decreasing the number of gastric carcinoma cells [150].

A broad study was also conducted on the role of adenovirus and the correlation of it with TIPE2 expression. TIPE2 is tumor necrosis factor-alpha induced protein 8-like 2 downregulating innate immunity via impacting on TLR signaling, macrophages and dendritic cells [151]. It was shown that TIPE2 is reduced or absent in several tumors, including gastric cancer [144,152]. TIPE2 is an inhibitor of gastric cancer cell growth, and might promote a p27-associated signaling cascade that leads to control the cell cycle and cell division, leading to the conclusion, that TIPE2 may regulate proliferation of gastric cells [152]. Moreover, according to Zhu et al. [146], TIPE2 may be a novel potential therapeutic target for human gastric cancer, on the basis of the results achieved in a panel of human gastric cells (AGS, HGC-27 and SGC-7901), where expression of TIPE2 was lost. Adenovirus-mediated human TIPE2 overexpression significantly inhibited AGS and HGC-27 gastric cancer cell growth and induced AGS and HGC-27 tumor cell apoptosis in vitro [146]. Further investigations showed [151], that adenovirus-mediated TIPE2 upregulate E-cadherin epithelial marker in AGS and HGC-27 in in vitro and in vivo model, leading to the conclusion, that TIPE2 not only inhibits gastric cancer cell migration, but also stating that invasion and metastasis in gastric cancer in probably via reversal of epithelial–mesenchymal transition, which may be crucial in further therapeutic approaches [151].

Adenovirus vector was also targeted at cancer associated fibroblasts (CAF), being the crucial microenvironment of tumor growth, invasion and metastasis [147]. CAFs contribute to cancer growth and metastasis by secreting cytokines, growth factors and adhesion molecules, leading to enhancement of radio and chemotherapy resistance, that is why CAFs seem to be a potentially successful target for cancer therapy via adenovirus vectors [147]. Such studies have been also performed in relation to gastric cancer, and it was concluded, that fiber-modified hexon-chimeric recombinant oncolytic adenovirus targeting CAFs can kill gastric CAFs and inhibit gastric cancer growth in vivo [147].

On the basis of the fact, that most cancer cells are characterized with an increased telomerase activity, studied have been performed with telomerase-specific oncolytic adenovirus, which can suppress tumor cells, not influencing the healthy ones [153]. This was also confirmed in studies on gastric cancer cells in vitro, by combining tumor-specific TRAIL (tumor necrosis factor-related apoptosis-inducing ligand) with adenovirus vector [146]. Moreover, this type of novel therapeutic approach was noted to be successful even in advance stage of gastric cancer with peritoneal dissemination [146].

## 5. Oncolytic Viruses

Oncolytic viruses are promising cancer gene therapy agents, as they have the ability to selectively replicate in cancer cells, causing cancer cell lysis and inflammation, leading to the stimulation of host immune responses to cancer cells [4].

Oncolytic viruses are successfully used in cancer immunotherapy, as they target multiple steps within the cancer-immunity cycle [154,155]. The ability of viruses to attack cancer cells was discovered in the mid-20th century [156,157], but the first clinical trials documenting the actual clinical benefits of using oncolytic viruses have been carried out over the last 15 years [154]. Currently, with increasing knowledge about viruses in general and the constant development of research and therapeutic techniques, the interest in viruses as factors used in cancer immunotherapy is constantly growing. Many research teams are working on the development of optimized therapy with viruses. At this point in time, the only oncolytic viruses approved for cancer therapy are: Talimogene laherparepvec (T-VEC) approved by the FDA in 2017 [158] and genetically modified adenovirus H101 approved in 2006 in China [159]. The former was approved as immunotherapy for patients with advanced melanoma, the latter for treating head and neck cancer. These are huge successes in cancer therapy and are likely to significantly contribute to the development of this field of immunotherapy. Some other trials are still ongoing, like the one using the measles virus TMV-018 (ClinicalTrials.gov.NCT04195373), or vaccinia viral oncolytic vector (GL-ONC1; ClinicalTrials.gov.NCT01443260).

The mechanism of action of oncolytic viruses may be different. They may lead to direct lysis of cancer cells, leading to the release of soluble antigens, danger signals and type I interferons, which drive antitumour immunity. Furthermore, some oncolytic viruses may be created artificially to express therapeutic genes. They can also alter tumor-related endothelial cells, which increases the recruitment of T lymphocytes into excluded or immunocompromised tumor microenvironments. Ultimately, oncolytic viruses can also be used as a source of in situ neoantigenic vaccinations through their cross-presentation, which leads to distant uninfected tumors [155].

These features make scientists willing to study the efficacy of oncolytic viruses. However, further studies are necessary to develop better therapies using them.

There are several oncolytic viruses (Table 3), and they are divided into two classes—firstly viruses that naturally replicate in cancer cells and are usually mild in human infection, such as parvoviruses, myxoma virus (MYXV), Newcastle disease virus (NDV), reovirus and Seneca valley virus (SVV) [160]. Second class contains viruses that are genetically changed and used as vectors, including measles virus (MV), poliovirus (PV) and vaccinia virus (VV) [160]. In this group, also genetically-engineered viruses are included, characterized by mutations in genes required for replication in normal conditions, and among such viruses, adenovirus, herpes simplex virus (HSV) and vesicular stomatitis virus (VSV) are enumerated [160]. The last group seems to be of special interest, not only due to the potential in cancer therapies, but also because of being a double-edged sword in this matter, and those viruses will be discussed, excluding adenovirus, placed as a puzzle between oncogenic and oncolytic viruses in gastric cancer.

### Oncolytic Viruses and the Immune System

**Figure 2:**
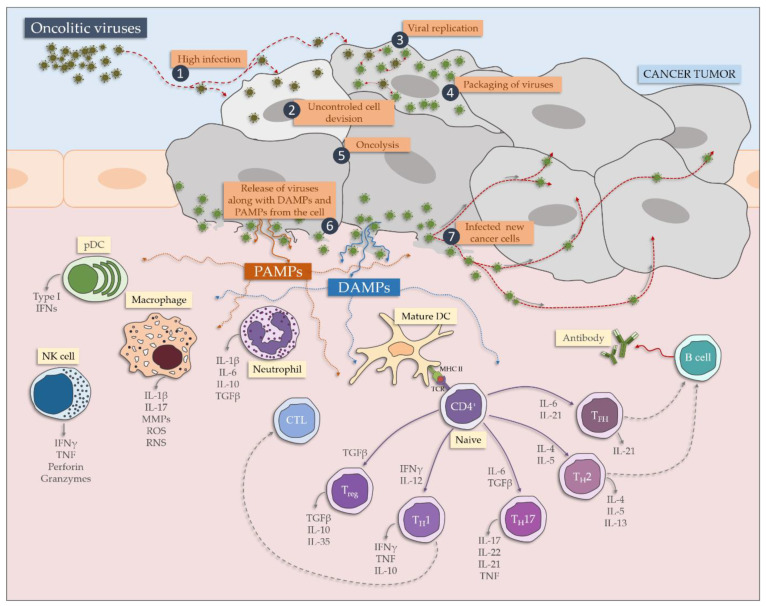
The impact on oncolytic viruses on the immune system.

During the infection with an oncolytic virus, a panel of immune cells is recruited, from both innate and adaptive immune signaling (Figure 2). After recognition by anti-viral PRRs (mainly TLRs and RIG-1), the production of pro-inflammatory cytokines and interferons takes place. Neutrophils and macrophages release several inflammatory mediators, cationic proteins, lipid mediators, metalloproteinases and components of oxygen burst [18]. Additionally, DC and NK (natural killer) are triggered and the virus is presented to the panel of T cells—T helper cells responses are induced, followed by CTL killing of the virally infected cells and causing tissue damage. Additionally, T_H_17 contribute to an inflammatory response. The process is impaired by Treg. Finally, B cells are activated to antibodies production [18].

## 6. Oncolytic Viruses in Gastric Cancer

### 6.1. Herpes Simplex Virus

HSV, a member of Herpesviridae [23], is an enveloped ds linear DNA virus, with genes classified into three groups by the regulation of their expression—immediate early (IE), early (E) and late (L) [174]. The IE gene products regulate gene transcription and include the US12 gene product, which is ICP47—responsible for silencing MHC I expression in infected cells via inhibition of TAP (transporter associated with antigen presentation) [174]. The E genes promote viral DNA synthesis and the L genes are coding capsid proteins, tegument proteins and envelope glycoproteins [174].

One of the advantages of using HSV in oncolytic virotherapy is the fact, that this virus can bind only to a single receptor, which gives the opportunity to use it in treatments of many malignancies, due to the existence of four cellular receptors on HSV [174]. Moreover, HSV, due to its large genome, is able to incorporate a large size of a foreign DNA, the infection may be quite easily controlled with anti-herpetic drugs and can kill target cell faster and more effectively comparing to adenovirus [174,175]. Among genes important for effective oncolysis, ICP0, ICP4 and ICP47 are enumerated, but it is worth adding, that some of this data is more than ten years old now [174].

There are several HSV strategies to avoid the host’s immune response, including complementing immunoglobulins via viral glycoproteins, inhibition of cytokine production, blocking the maturation of APC (antigen presenting cells), expression of MHC II, inhibiting apoptosis and cell death induced by CTL (cytotoxic lymphocytes) [175].

Several oncolytic mutants of HSV are in different stages of clinical trials on solid tumors with a high level of success [176]. However, their efficacy depends on the extent of both intratumoral viral replication and induction of a host antitumor immune response [175]. Such an immune response may induce the upregulation of angiogenic factors and downregulation of antiangiogenic factors, such as thrombospondin-1 (TSP-1), but some moderations are made to increase the oncolytic action of the virus [176]. In studies of Tsuji et al. [176], replication-competent oncolytic HSV was constructed as a vector to deliver TSP-1 to a gastric cancer microenvironment, and this enhanced antitumor efficacy in vitro and in vivo via direct antitumor and antiangiogenic mechanisms. Moreover, in gastric tumor cell line SGC7901, the synergistic antitumor effect of herpes virus thymidine kinase (HSV-TK) with TNF-α and IL-2 gene expression was evaluated, but with no significant effect [177]. On the other hand, other studies on mice model showed a therapeutic effect of HSV-TK expression [178], so further studies are needed in this matter.

Additionally, in gastric cancer, two multimutant oncolytic herpes simplex viruses of the second generation—G207 and NV1020—have been shown to kill in vitro human gastric cancer cells [179]. With the use of a murine xenograft model of peritoneally disseminated gastric cancer, it was registered, that with lower viral dose NV1020 was more effective comparing to G207, but intraperitoneal administration was crucial for the positive effect [179]. Those safe for animal pre-clinical trials are a promising pathway for successful treatment of gastric cancer. Additionally, studies were conducted in which G207 was combined with mitomycin C (MMC) and significant synergism was observed [180]. This combination upregulated GADD34 in tumor and thus may complement the gamma134.5 gene deletion in gastric human cells in vitro [180].

Moreover, preclinical trials have been conducted with the use of third generation oncolytic HSV-1–G47∆, which is a triple mutated virus developed by adding another deletion mutation to the genome of a second-generation HSV-1—G207 [181]. The use of this virus in GC (gastric cancer) decreased the level of M2 macrophages and increased the level of M1 macrophages and NK cells [181]. Interestingly, a strong antiviral response was reported leading to a controversial conclusion that innate immunity stimulated by oncolytic virus treatment may facilitate the priming of antitumor immunity [181].

### 6.2. Vesicular Stomatitis Virus (VSV)

Vesicular stomatitis virus, a member of the Rhabdoviridae family [23], is known to be replicating to induce apoptosis of many types of cells, including cancer cells, but only one report exists on the role of VSV in gastric cancer [182]. In gastric carcinoma cell line MKN28, the expression of vesicular stomatitis virus matrix protein (MP) was confirmed to inhibit proliferation and induce apoptosis on this type of cancer cell [182].

### 6.3. Vaccinia Virus

Vaccinia virus, a member of the Poxviridae family [23], may also be an attractive potential oncolytic virus for GC treatment, as stated above, even a clinical trial is ongoing—phase I and II completed, with the use of this virus (NCT01443260). Among the advantages of this virus, as far as genetic engineering is concerned, one can enumerate the ability to incorporate large amounts of foreign DNA without losing the replication efficiency and high safety in humans [183]. GLV-1 h153, which is a genetically engineered vaccinia virus carrying the human sodium iodide symporter (hNIS) gene, was tested as a potential and novel therapy against GC [183]. It was shown [183] that GLV-1 j153 is an effective oncolytic virus giving successful results in five human gastric cancer lines, giving over 90% cytotoxicity. This promising result may also be enhanced by the combination of treatment with GLV-1 h153 and radioiodine, which needs to be further studied [183].

## 7. Conclusions

Searching for an efficient and effective cancer treatment is one of the key interests in today’s world. Gastric cancer, no matter the downward trend, is still a major concern, and expanding treatment possibilities is a pivotal issue. Oncolytic virotherapy is surely one of the options. On the other hand, oncogenic role of viruses has been also known and is proven in gastric cancer. Showing a double-edge sword face of viruses in gastric cancer aimed at drawing the attention to a cautious choice of cancer treatment.

## Figures and Tables

**Table 1:** Human oncogenic virus.

Family	Virus	Cancer Type	References
**1. DNA viruses**			
**Hepadnaviridae**	HBV	Hepatocellular carcinoma, cholangiocarcinoma *, non-Hodgkin lymphoma *, gastric cancer *	[5,6,7,8,9,10,11,12]
**Herpesviridae**	EBV/HHV-4	Nasopharyngeal carcinoma, Burkitt lymphoma, immune-suppression-related non-Hodgkin lymphoma, extranodal natural killer/T-cell lymphoma (nasal type), posttransplant lymphoproliferative disorder, Hodgkin lymphoma, breast cancer *, gastric cancer *, leiomyosarcomas *, AIDS-associated lymphomas *	[5,6,7,8,9,10,11,12,13,14,15]
	KSHV/HHV-8	Kaposi sarcoma, primary effusion lymphoma, AIDS-related lymphoproliferative disorder *, multicentric Castleman’s Disease *	[5,6,7,8,9,10,11,12]
**Papillomaviridae**	HPV	Cervical cancer, oropharyngeal cancers, anal cancer, penile cancer, vaginal cancer, vulvar cancer, larynx cancer *	[5,6,8,9,10,11,12,13,16,17]
**2. RNA viruses**			
**Flaviviridae**	HCV	Hepatocellular carcinoma, non-Hodgkin’s lymphoma, cholangiocarcinoma *,	[5,6,7,8,9,10,11,12]
**Retroviridae**	HTLV-1	Adult T-cell leukemia/lymphoma (ALT)	[5,6,7,8,9,10,11,12]

HBV: Hepatitis B virus; EBV/HHV-4: Epstein–Barr virus/Human herpesvirus 4; KSHV/HHV-8: Kaposi’s sarcoma-associated herpesvirus/Human herpesvirus 8; HPV: Human Papillomavirus; HCV: Hepatitis C virus; HTLV-1: Human T-lymphotropic virus-1. * Cancer sites with limited evidence.

**Table 2:** Potentially oncogenic human viruses.

Family	Virus	Cancer Type	References
**1. DNA viruses**			
**Adenoviridae**	HAdV-A 12, 18, 31 HAdV-D 9	Various solid tumors in rodents	[5,9]
**Papovaviridae**	MCV/MCPyV	Merkel cell carcinoma	[5,6,7,11,16]
	JCV, BKV	Solid tumors in rodents and primates	[5,9,11]
**2. RNA viruses**			
**Retroviridae**	HIV-1	Kaposi’s sarcoma, non-Hodgkin lymphoma, Hodgkin’s lymphoma, cervical cancer, anal cancer, conjunctival cancer, vulvar cancer *, vaginal cancer *, penile cancer *, non melanoma skin cancer *, hepatocellular carcinoma	[5,11]
	HIV-2	Kaposi’s sarcoma *, non-Hodgkin’s lymphoma *	[11]
	HERV-K	Breast cancer	[11]
	XMRV	Prostate cancer	[11]

HAdV-A: Human Adenovirus A; HAdV-D: Human Adenovirus D; MCV/MCPyV: Merkel cell polyomavirus; JCV: JC polyomavirus; BKV: BK polyomavirus; HIV-1: Human immunodeficiency virus 1; HIV-2: Human immunodeficiency virus 2; HERV-K: Human endogenous retrovirus K; XMRV: Xenotropic murine leukemia virus-related virus. * Cancer sites with limited evidence.

**Table 3:** Oncolytic viruses.

Family	Virus	References
**1. DNA viruses**		
**Adenoviridae**	Adenovirus ^1^	[161,162,163,164,165,166,167,168]
**Herpesviridae**	Herpes simplex virus ^2^	[161,162,163,164,167,168]
**Parvoviridae**	Parvovirus	[163,168,169,170]
**Poxviridae**	Myxoma virus	[168,171]
	Vaccinia virus	[161,162,163,164,167,168,171]
**2. RNA viruses**		
**Orthomyxoviridae**	Influenza virus	[168,172]
**Paramyxoviridae**	Measles virus	[161,162,163,164,167,168,171,172]
	Mumps virus	[161,167,172,173]
	Newcastle disease virus	[161,162,163,164,166,168,170,172]
**Picornaviridae**	Coxsackievirus	[161,163,166,167,168,171]
	Echovirus	[161,166,167]
	Encephalomyocarditis virus and Mengovirus	[167]
	Enterovirus	[166,167]
	Seneca valley virus	[163,165,166,167]
	Theiler’s Murine Encephalomyelitis Virus	[167]
**Reoviridae**	Reovirus	[161,162,163,164,166,167,168,170,171,172]
**Retroviridae**	Retrovirus	[161,162,172]
**Rhabdoviridae**	Maraba virus	[161,163,169]
	Vesicular stomatitis virus	[161,162,163,164,167,168,172]

1—approved in China; 2—approved in USA.
